# High-Pressure ESR Spectroscopy: On the Rotational Motion of Spin Probes in Pressurized Ionic Liquids

**DOI:** 10.1021/acs.jpcb.1c09243

**Published:** 2022-01-24

**Authors:** Boryana Y. Mladenova Kattnig, Daniel R. Kattnig, Guenter Grampp

**Affiliations:** † Living Systems Institute, University of Exeter, Stocker Road, Exeter, EX4 4QD, U.K.; ‡ Institute of Physical and Theoretical Chemistry, Graz University of Technology, Stremayrgasse 9, A-8010 Graz, Austria

## Abstract

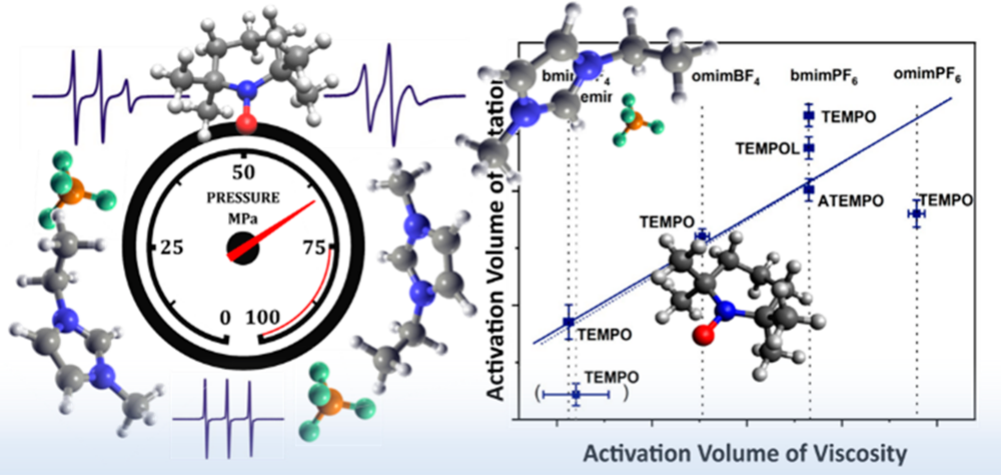

We report high-pressure (up to 50 MPa) ESR-spectroscopic investigations on the rotational correlation times of the nitroxide radicals 2,2,6,6-tetramethylpiperidine 1-oxyl (TEMPO), 4-hydroxy-2,2,6,6-tetramethylpiperidine 1-oxyl (TEMPOL), and 4-amino-2,2,6,6-tetramethylpiperidine 1-oxyl (ATEMPO) in the ionic liquids 1-ethyl-3-methylimidazolium tetrafluoroborate (emimBF_4_), 1-butyl-3-methylimidazolium hexafluorophosphate (bmimPF_6_), 1-butyl-3-methylimidazolium tetrafluoroborate (bmimBF_4_), 1-methyl-3-octylimidazolium tetrafluoroborate (omimBF_4_), and 1-methyl-3-octylimidazolium hexafluorophosphate (omimPF_6_). The activation volumes (38.5–56.6 Å^3^) determined from pressure dependent rotational diffusion coefficients agree well with the pressure dependent viscosities of the ionic liquids. Experimentally, the fractional exponent of the generalized Stokes–Einstein–Debye relation is found to be close to one.

## Introduction

Room temperature ionic liquids (RTILs) exhibit a larger number of unusual physical properties, making them attractive as solvent media for academic and industrial applications.^1−13^ Beside their well-known physical properties such as an extremely low vapor pressure, wide electrochemical windows, and high thermal stability, recently astonishing nanostructured effects have been reported, like the formation of nano- to mesoscaled polar and unpolar domains. Peric et al. reported in a detailed study about the nanostructured organization of RTILs using perdeuterated TEMPONE radical as a spin probe.^14^ Only two papers deal with time-resolved (TR) ESR-spectroscopy. Kawai et al. and recently Fedin et al. report on the spin dynamics of triplet and doublet states in RTILs.^15,16^

High-field ESR-spectroscopic studies support the assumption that polar domains are formed by anions and cations whereas the unpolar domains are formed by the alkyl chains of the RTILs. Using charged and uncharged nitroxide radicals, several authors reported different correlation times based on different anions in the RTILs.^17−19^

Interactions between imidazolium-based ionic liquids and some nitroxyl radicals carrying different substituents, such as hydrogen bonding −OH or ionic −N(CH_3_)^3+^ and −OSO^3–^ substituents have been systematically investigated by Zhang et all.^20^ Their studies based on density functional theory calculations predict significant reduction of the mobility of ionic radicals in ionic liquids compared to systems containing neutral radicals.

The study of diffusion influenced reactions is of great interest not only because of the generally high viscosity of the RTILs but also from the viewpoint of solute–solvent cage and microenvironmental effects.^21−23^

The rotational mobility of organic radicals in conventional solvents and in room temperature ionic liquids sensed by ESR-spectroscopy has been subject of several articles published by Feed, Strehmel, Evans, and Kawai.^24−33^ The first study on rotational motion in RTILs was published by Allendoerfer et al. in 1992.^34^

Rotational motion of spin probes in imidazolium-based ionic liquids has been reported by Sengupta and Miyake as well.^35−37^

Studies on pressure-dependent ESR-line width and rotational correlation times of vandyl acetylacetonate in variety of nonhydrogen-bonded solvents have been reported by Hwang at al.^38^ Recently Hubbel et al. reintroduced the application of high-pressure ESR spectroscopy to biochemical reactions in water. They report on protein-folding, conformational equilibria of spin-labeled proteins.^39−41^

For the study of the 3-carboxypropyl radical, disagreeing rates of rotational diffusion and the corresponding activation energies with those calculated from the Stokes–Einstein–Debye (SED) relation have been found. Often a fractional dependence on (η/*T*)^*x*^ is suggested with *x* < 1 where η and *T* denote the dynamic viscosity and the temperature, respectively. Such corrections could indicate a slippage of the solute in the solvent cage. The microviscosity model introduced by Gierer and Wertz is mainly used for such deviations.^42^

In contrast to these findings, Evans et al. have reported on the activation energy of rotational diffusion of TEMPO, which correlates well with the activation energies of the viscous flow of the RTILs as predicted by the classical SED-relation.^25^

Strehmel at al. reported on the influence of the alkyl chain length of the RTILs on the rotation of 4-hydroxy-2,2,6,6-tetramethyl-1-piperidinyloxy (TEMPOL) radical. A linear correlation of the rotational correlation times with the viscosity is also reported by these authors. For charged spin probes, deviations from the SED-behavior are found.^26^

We recently studied the temperature dependence of several spin probes in RTILs and found good correlation with the SED-equation, albeit too small hydrodynamic radii, even if deviations from the spherical shape of the spin probes and microviscosity corrections are taken into account.^21,23,43−45^

All these studies reported in the literature focused on viscosity and/or temperature dependent measurements. To get insights into the free-volume effect, the formation and coalescence of specific domains in RTILs, additional pressure dependent investigations appear worthwhile. Here, we report for the first time on pressure dependent measurements of rotational correlation times undertaken with the aim of revealing detailed information on the corresponding activation volumes, Δ*V*^‡^.

## Methods

CW-ESR spectra were recorded using a JEOL PE-3X spectrometer equipped with an improved microwave bridging system and an AEG magnet. A cylindrical TEM_011_-cavity was used. The spectrometer operated at a microwave frequency of around 9.5 GHz and employed a 100 kHz field modulation. A home build flow-through sample cell system, allowing measurements at elevated pressures of up to 100 MPa is described elsewhere.^46,47^

**Scheme 1:**
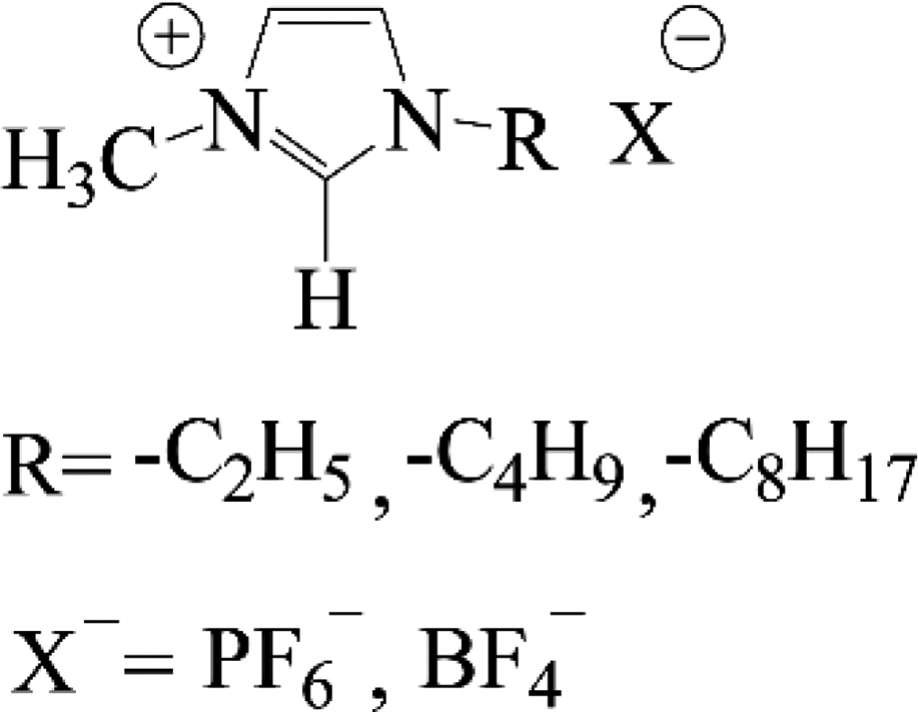
Ionic Liquids Used

The ionic liquids 1-ethyl-3-methylimidazolium tetrafluoroborate (emimBF_4_, > 98%), 1-butyl-3-methylimidazolium hexafluorophosphate (bmimPF_6_, > 99%), and 1-butyl-3-methylimidazolium tetrafluoroborate (bmimBF_4_,>99%) were purchased from Ionic Liquids Technologies (IoLiTec, Germany). 1-methyl-3-octylimidazolium tetrafluoroborate (omimBF_4_, > 98%) and 1-methyl-3-octylimidazolium hexafluorophosphate (omimPF_6_, > 98%) were obtained from Solchemar, Portugal. Structures of the studied RTILs are shown in Scheme 1

On account of the fact that small traces of water can change significantly the rotational correlation times of the spin labels, all RTILs were dried for at least 24 h under high vacuum (<5 × 10^–5^ Torr) and at elevated temperatures (327–337 K). The dried ionic liquids were stored in Schlenk tubes under an argon atmosphere. The tubes were furthermore placed in an desiccator over P_4_O_10_ and kept in dark.

**Figure 1:**
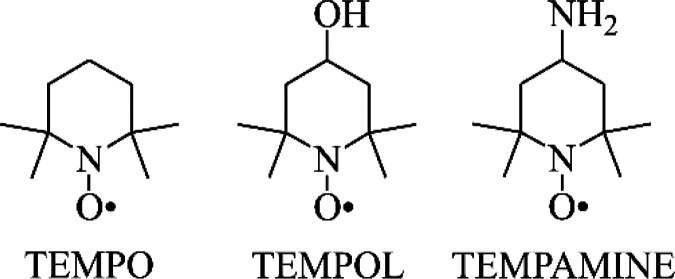
Used spin labels

Used spin labels (see Figure 1) 2,2,6,6-tetramethylpiperidine 1-oxyl (TEMPO, > 99%) and 4-amino-2,2,6,6-tetramethylpiperidine-1-oxyl (ATEMPO, > 97%) were obtained from Sigma-Aldrich. Before use, TEMPO was purified by sublimation. 4-Hydroxy-2,2,6,6-tetramethylpiperidine 1-oxyl, (TEMPOL, ≥ 97%,) was purchased from Fluka and used as received.

The volume of the RTILs was determined gravimetrically from the density of the corresponding solvent. In order to avoid line broadening effects, due to spin–spin interactions, the radical concentrations were kept low at 3 × 10^–4^ M to 5 × 10^–4^ M. Standard Schlenk techniques were employed to transfer the sample solutions under argon atmosphere into the ESR-spectrometer. All samples were measured at 295 ± 1 K. Prior each measurement at least 15 min were allowed for thermostatting. The pressure was increased from 0.1 to 50 MPa following reduction to 0.1 MPa in steps of 2.5 MPa. No hysteresis was found for all measurements. EPR spectra were simulated using EasySpin toolbox for Matlab.^47,48^ The *g*- and *A*-tensors were determined from measurements of the corresponding sample at 80 K using a Bruker E580-FF/CW spectrometer by courtesy of the Ruđer Bošković Institute (Zagreb, Croatia), equipped with a liquid helium/nitrogen cryostat (Oxford Instruments).^43,44^ The spin-Hamiltonian parameters of the investigated sin probes in the studied ILs used for the simulation of the experimental EPR spectra are listed in Table 1.

**Table 1:** Spin-Hamiltonian Parameters of the Investigated Sin Probes in the Studied ILs

solvent	substance	*g*_*xx*_	*g*_*yy*_	*g*_*zz*_	*A*_*xx*_/MHz	*A*_*yx*_/MHz	*A*_*zz*_/MHz
emimBF_4_	TEMPO	2.0111	2.0089	2.0044	44.8	27.5	–5.9
bmimBF_4_	TEMPO	2.0087	2.0065	2.002	44.8	27.5	–5.9
bmimPF_6_	TEMPO	2.0087	2.0065	2.002	44.8	27.5	–5.9
bmimPF_6_	TEMPOL	2.0109	2.0089	2.0043	43.8	27.6	–7.0
bmimPF_6_	ATEMPO	2.0111	2.009	2.0045	44.9	27.3	–7.8
omimBF_4_	TEMPO	2.0087	2.0065	2.002	44.0	27.5	–5.9
omimPF_6_	TEMPO	2.0087	2.0065	2.002	44.4	27.5	–5.9

## Results and Discussion

**Figure 2:**
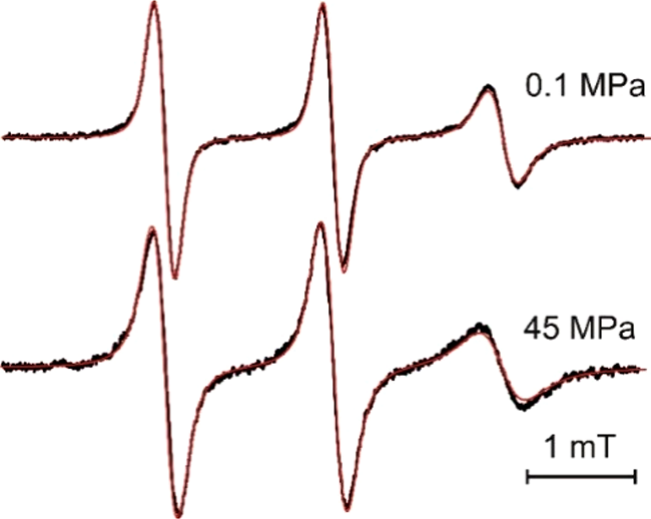
Pressure-dependent EPR spectra of TEMPOL in bmimPF_6_ at 295 K (experimental black and simulated red line). The rotational rate constants extracted from the simulations are *k*_*r*_ = 2.75 × 10^7^ s^–1^ and 5.74 × 10^7^ s^–1^ from bottom to the top, respectively.

In this study we focus on fast tumbling spin probes (see Figure. 1). Typical ESR-spectra recorded at atmospheric and elevated pressure, together with their computer simulations, are shown on Figure. 2.

At constant temperature, pressure dependent rate constants *k*_*r*_ result in the corresponding experimental activation volumes ΔV‡_obs_.
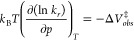
1

The Stokes–Einstein–Debye (SED) law is routinely used in the literature, to relate rotational diffusion coefficients to solvent viscosities, eq 2.
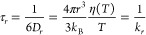
2

In this expression, τ_r_ is the rotational correlation time, *D*_*r*_ the rotational diffusion coefficient, and η the dynamic viscosity of the solvent. *r* is an effective hydrodynamic radius that is expected to correspond or exceed the van der Waals radius of the spin probe. Often the experimental *r* deviates from the van der Waals one and is then denoted as hydrodynamic radius. *k*_*B*_ and *T* denote the Boltzmann constant and the absolute temperature. Deviations from SED-relation are normally expressed by a fractional SED-expression with exponent *x*:
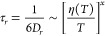
3The viscosities of the studied ionic liquids at room temperature and atmospheric pressure are listed in Table 2.

**Table 2:** Viscosities of the Studied Ionic Liquids at 295 K and *P* = 0.1 MPa

solvent	η/(mPa s)
emimBF_4_^35^	43
bmimBF_4_^36^	122
bmimPF_6_^37^	327
omimBF_4_^38^	417
omimPF_6_^38^	919

Viscosities at elevated pressures are obtained from published data^2,11−13^ and were fitted to an Arrhenius type equation, eq 4.
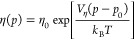
4

The fitted parameters ε_0_ and *V*_η_/*k*_*B*_*T* are given in Table 3.

**Table 3:** Coefficients of Best Fits Using eq 4^a^

Solvent	η_0_	*V*_η_/*k*_*B*_*T*
emimBF_4_^2^	43.34	0.010
bmimBF_4_^12^	112.23	0.001
bmimPF_6_^11^	284.36	0.013
omimBF_4_^13^	353.12	0.012
omimPF_6_^13^	733.14	0.014

a See refs (2, 11−13, 49, and 50).

**Figure 3:**
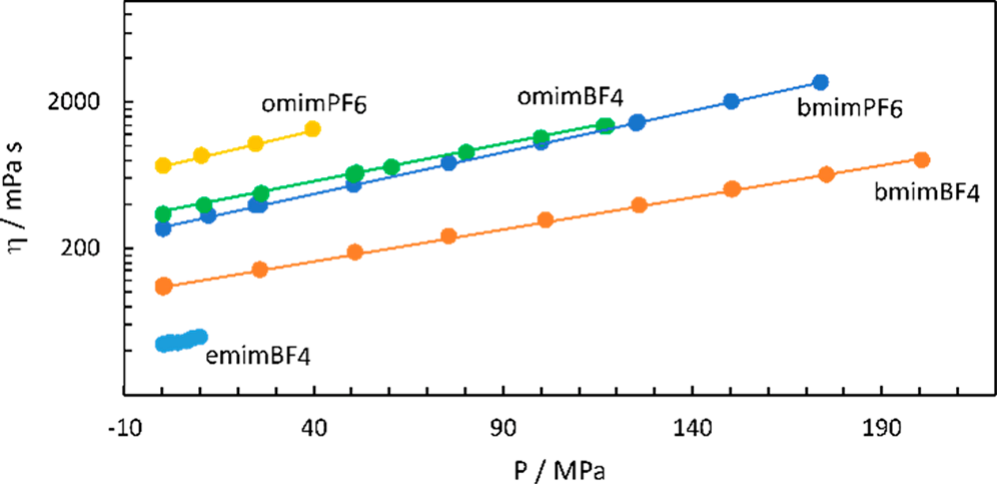
Pressure dependence on the viscosity of the studied room temperature ionic liquids.

The corresponding volumes *V*_η_, given in Table 3 have been extracted from plots like that given in Figure 3.

**Figure 4:**
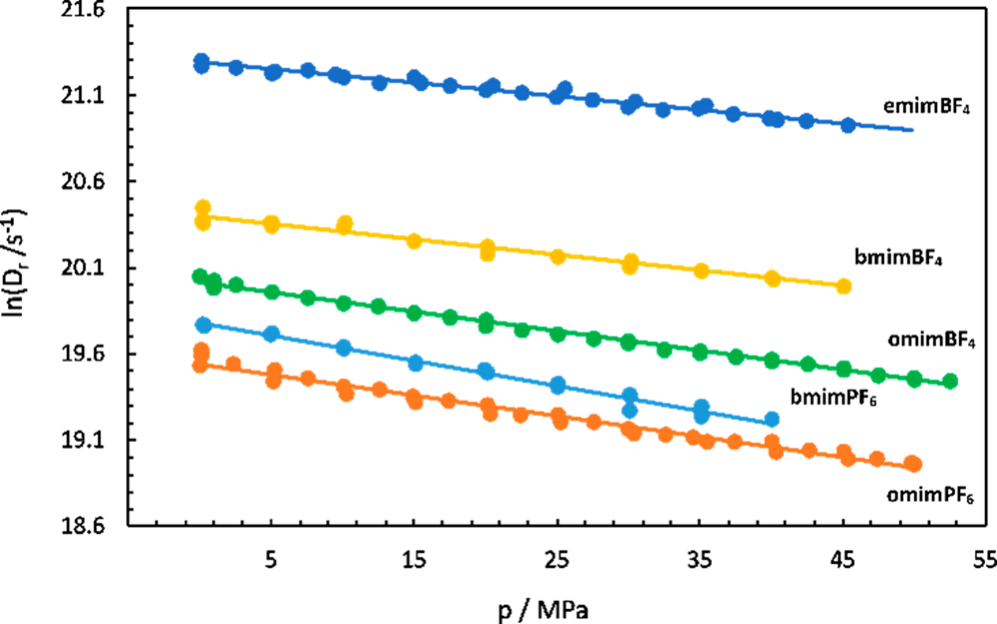
Rotational diffusion coefficients of TEMPO as a function of pressure at *T* = 295 K.

From the pressure dependence of the rotational diffusion coefficients, listed in Table 4 and presented on Figure 4, the experimental activation volumes, Δ*V*_obs_^‡^, given in Table 5 were extracted.

**Table 4:** Rotational Diffusion Coefficients of Various Spin Probes in the Studied Room Temperature Ionic Liquids at Atmospheric and Elevated Pressures, *T* = 295 K

		*D*_*r*_ × 10^8^/s	
substance	solvent	0.1 MPa	50 MPa
TEMPO	emimBF_4_	17.3	12.7
TEMPO	bmimBF_4_	7.61	6.96
TEMPO	bmimPF_6_	3.88	1.93
TEMPO	omimBF_4_	4.91	2.83
TEMPO	omimPF_6_	3.04	1.71
TEMPOL	bmimPF_6_	3.09	1.53
ATEMPO	bmimPF_6_	1.41	0.77

**Table 5:** Activation Volumes

RTIL	substance	*V*_η_/Å^3^	Δ*V*_*obs*_^‡^/Å^3^	ΔΔ*V*/%
bmimBF_4_	TEMPO	40.6 ± 0.3	38.5 ± 1.5	–5
omimBF_4_	TEMPO	47.6 ± 0.4	46.0 ± 0.6	–3
omimPF_6_	TEMPO	58.9 ± 0.4	48.0 ± 1.2	–19
emimBF_4_	TEMPO	41.0 ± 1.7	32.2 ± 1.0	–22
bmimPF_6_	TEMPO	53.2 ± 0.2	56.6 ± 1.0	6
bmimPF_6_	TEMPOL	53.2 ± 0.2	53.8 ± 1.0	1
bmimPF_6_	ATEMPO	53.2 ± 0.2	50.1 ± 1.0	–6

For all samples, the rotational diffusion coefficients agreed upon increasing and decreasing the pressure. No hysteresis was apparent.

Combining eqs 1, 2, and 3 and differentiating with respect to pressure leads to the following expression for the activation volumes:
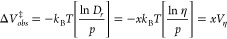
5This linear relation combines both experimental quantities *ΔV*_*obs*_^‡^ and *V*_η_. The slope of such a plot is directly given by the exponent *x* of the fractional SED-relation, an empirical quantity normally obtained by fitting experimental and the theoretical SED-relations.

**Figure 5:**
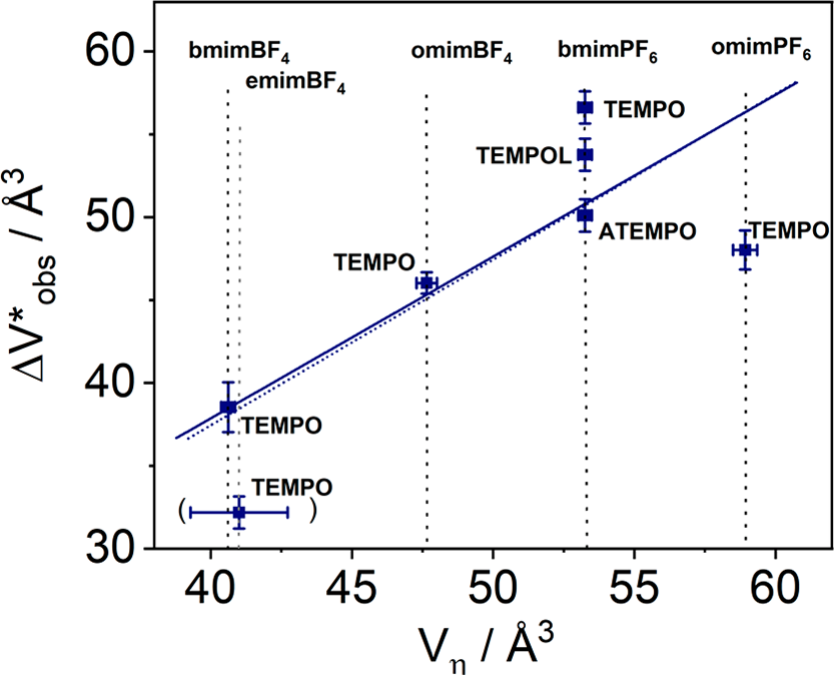
Plot of *ΔV*_*obs*_^‡^ vs *V*_η_ for the studied RTILs and spin labels. Dashed line corresponds to a slope of 1.

Figure 5 shows a plot according to eq 5 for TEMPO, TEMPOL, and ATEMPO in different RTILs. A slope of 1.1 ± 0.3 is found for the TEMPO probe in the investigated RTILs, and a slope of 1.0 ± 0.1 is found when all investigated substances and ILs are taken in consideration. This result indicates that in the frame of the experimental error there are no significant deviation from the SED behavior in pressurized ILs. A fractional dependence would result in *ΔV*_*obs*_^‡^ < *V*_η_ and *x* < 1. Investigations on temperature-dependent phase transitions, which may be also pressure dependent, are reported.^51−53^

The quantity ΔΔ*V* presented in Table 4 gives the relative deviation of *ΔV*_*obs*_^‡^ from *V*_η_. For TEMPO ΔΔV does not correlate with viscosity η (see Table 4). For various probe molecules in bmimPF_6_, ΔΔV decreases with increasing acceptor strength of the 4-substituent. For ATEMPO, a beginning decoupling of η and τ_*r*_ can be realized, which leads to a pronounced fractional SED-behavior. This is a likely consequence of the of the acceptor properties of the amino-group in the 4-position.

The agreement between experimental rotational correlation times and predictions based on the Stokes–Einstein–Debye relation appears surprising in view of the combined scientific literature so far. Many publications have documented deviations from Stokes–Einstein and Stokes–Einstein–Debye (SED) relations in pure ionic liquids and ionic liquid/solvent mixtures. Often these deviations are ascribed to strong solvent–solute interactions and/or differences in size and shape of the RTIL constituents and the molecules investigated.^54−59^ On the other hand, the SED is based on continuum hydrodynamics and applies to large solutes immersed in a homogeneous environment. In fact, already Einstein pointed out that there is little reason to apply SED-like equations on the molecular scale.^60^ In view of these observations, our findings of SED-behavior in pressurized room temperature ionic liquids are unforeseen, both generally and in the present RTIL context, for which RTIL constituents and spin probes are clearly of comparable size and strongly interacting.

## Conclusions

In this study, we have shown for first time that the rotational correlation times for the spin labels TEMPO, TEMPOL, and ATEMPO in several imidazolium-based ionic liquids depend linearly on the pressure. The Stokes–Einstein–Debye relation is fulfilled, which means the correlation times τ_*r*_ are directly proportional to the viscosity η of the RTILs and no fractional correction of the SED-relation is necessitated. From experimental plots of *V*_*obs*_ versus *V*_η_, *x* is found to be equal to 1. For the TEMPO radical, the quantity ΔΔ*V* (%) is not related to the RTIL viscosity η. In bmimPF_6_, ΔΔ*V* decreases with increasing acceptor properties of the substituent in the 4-position. For ATEMPO, the viscosity η increases more strongly with pressure than the correlation time τ_*r*_, indicating the beginning decoupling of η and τ_*r*_, leading to a fractional SED-behavior.

## Abbreviations

SED: Stokes–Einstein–Debye
ESR: electron spin resonance
RTIL: room temperature ionic liquid
TEMPO: 2,2,6,6-tetramethylpiperidine 1-oxyl
TEMPOL: 4-hydroxy-2,2,6,6-tetramethylpiperidine 1-oxyl
ATEMPO: 4-amino-2,2,6,6-tetramethylpiperidine 1-oxyl

## References

[ref1] Grampp G.; Kattnig D. R.; Mladenova B.; Rasmussen K. Chapter 4. 3 EPR Spectroscopy in Room Temperature Ionic Liquids. In Electron Paramagnetic Resonance; Chechik V., Murphy D. M., Gilbert B., Eds.; Royal Society of Chemistry: Cambridge, 2014; Vol. 24, pp 77–101; 10.1039/9781782620280-00077.

[ref2] Sanmamed Y. A.; González-Salgado D.; Troncoso J.; Romani L.; Baylaucq A.; Boned C. Experimental Methodology for Precise Determination of Density of RTILs as a Function of Temperature and Pressure Using Vibrating Tube Densimeters. J. Chem. Thermodyn. 2010, 42 (4), 553–563. 10.1016/j.jct.2009.11.014.

[ref3] Samanta A. Fluorescence Probing of the Physicochemical Characteristics of the Room Temperature Ionic Liquids. In Advanced Fluorescence Reporters in Chemistry and Biology III; Demchenko A. P., Ed.; Springer Series on Fluorescence; Springer Berlin Heidelberg: Berlin, Heidelberg, 2011; Vol. 113, pp 65–89; 10.1007/978-3-642-18035-4_2.

[ref4] Ionic Liquids Uncoiled: Critical Expert Overviews; Plechkova N. V., Seddon K. R., Eds.; Wiley: Hoboken, N.J, 2013.

[ref5] Wasserscheid P.; Keim W. Ionic Liquids-New “Solutions” for Transition Metal Catalysis. Angew. Chem., Int. Ed. Engl. 2000, 39 (21), 3772–3789. 10.1002/1521-3773(20001103)39:21<3772::AID-ANIE3772>3.0.CO;2-5. 11091453

[ref6] Kokorin A. Kinetics and Mechanism of Reactions of Aliphatic Stable Nitroxide Radicals in Chemical and Biological Chain Processes.; INTECH Open Access Publisher: 2012.

[ref7] Bankmann D.; Giernoth R. Magnetic Resonance Spectroscopy in Ionic Liquids. Prog. Nucl. Magn. Reson. Spectrosc. 2007, 51 (1), 63–90. 10.1016/j.pnmrs.2007.02.007.

[ref8] Turro N. J.; Chen J. Y.-C. Nitroxides. Applications in Chemistry, Biomedicine, and Materials Science. By Gertz L. Likhtenshtein, Jun Yamauchi, Shin′ichi Nakatsuji, Alex I. Smirnov, and Rui Tamura. Angew. Chem., Int. Ed. 2008, 47 (50), 9596–9597. 10.1002/anie.200885605.

[ref9] Hallett J. P.; Welton T. Room-Temperature Ionic Liquids: Solvents for Synthesis and Catalysis. 2. Chem. Rev. 2011, 111 (5), 3508–3576. 10.1021/cr1003248. 21469639

[ref10] Welton T. Room-Temperature Ionic Liquids. Solvents for Synthesis and Catalysis. Chem. Rev. 1999, 99 (8), 2071–2084. 10.1021/cr980032t. 11849019

[ref11] Harris K. R.; Woolf L. A.; Kanakubo M. Temperature and Pressure Dependence of the Viscosity of the Ionic Liquid 1-Butyl-3-Methylimidazolium Hexafluorophosphate. J. Chem. Eng. Data 2005, 50 (5), 1777–1782. 10.1021/je050147b.

[ref12] Harris K. R.; Kanakubo M.; Woolf L. A. Temperature and Pressure Dependence of the Viscosity of the Ionic Liquid 1-Butyl-3-Methylimidazolium Tetrafluoroborate: Viscosity and Density Relationships in Ionic Liquids. J. Chem. Eng. Data 2007, 52 (6), 2425–2430. 10.1021/je700370z.

[ref13] Harris K. R.; Kanakubo M.; Woolf L. A. Temperature and Pressure Dependence of the Viscosity of the Ionic Liquids 1-Methyl-3-Octylimidazolium Hexafluorophosphate and 1-Methyl-3-Octylimidazolium Tetrafluoroborate. J. Chem. Eng. Data 2006, 51 (3), 1161–1167. 10.1021/je060082s.

[ref14] Merunka D.; Peric M.; Peric M. Study of Nanostructural Organization of Ionic Liquids by Electron Paramagnetic Resonance Spectroscopy. J. Phys. Chem. B 2015, 119 (7), 3185–3193. 10.1021/jp512487y. 25594422 PMC4871252

[ref15] Ivanov M. Yu; Veber S. L.; Prikhod’ko S. A.; Adonin N. Yu; Bagryanskaya E. G.; Fedin M. V. Probing Microenvironment in Ionic Liquids by Time-Resolved EPR of Photoexcited Triplets. J. Phys. Chem. B 2015, 119 (42), 13440–13449. 10.1021/acs.jpcb.5b06792. 26421723

[ref16] Kawai A.; Hidemori T.; Shibuya K. Electron Spin Dynamics of Triplet and Doublet Molecules in Room Temperature Ionic Liquids Studied by a Time-Resolved EPR Method. Mol. Phys. 2006, 104 (10–11), 1573–1579. 10.1080/00268970500513602.

[ref17] Kattnig D. R.; Hinderberger D. Temperature-Dependent Formation and Transformation of Mesostructures in Water-Ionic Liquid Mixtures. Chem. - Asian J. 2012, 7 (5), 1000–1008. 10.1002/asia.201101040. 22422609

[ref18] Kattnig D. R.; Akdogan Y.; Lieberwirth I.; Hinderberger D. Spin Probing of Supramolecular Structures in 1-Butyl-3-Methyl-Imidazolium Tetrafluoroborate/Water Mixtures. Mol. Phys. 2013, 111 (18–19), 2723–2737. 10.1080/00268976.2013.793420.

[ref19] Akdogan Y.; Heller J.; Zimmermann H.; Hinderberger D. The Solvation of Nitroxide Radicals in Ionic Liquids Studied by High-Field EPR Spectroscopy. Phys. Chem. Chem. Phys. 2010, 12 (28), 7874 10.1039/c001602k. 20502835

[ref20] Zhang S.; Wang G.; Lu Y.; Zhu W.; Peng C.; Liu H. The Interactions between Imidazolium-Based Ionic Liquids and Stable Nitroxide Radical Species: A Theoretical Study. J. Phys. Chem. A 2016, 120 (30), 6089–6102. 10.1021/acs.jpca.6b05770. 27428048

[ref21] Pergushov V. I.; Chumakova N. A.; Mel’nikov M. Ya; Grampp G.; Kokorin A. I. Structural and Dynamic Microheterogeneity of Ionic Liquid. Dokl. Phys. Chem. 2009, 425 (2), 69–72. 10.1134/S0012501609040010.

[ref22] Stoesser R.; Herrmann W.; Zehl A.; Laschewsky A.; Strehmel V. Microviscosity and Micropolarity Effects of Imidazolium Based Ionic Liquids Investigated by Spin Probes Their Diffusion and Spin Exchange. Z. Für Phys. Chem. 2006, 220 (10), 1309–1342. 10.1524/zpch.2006.220.10.1309.

[ref23] Chumakova N. A.; Pergushov V. I.; Vorobiev A. Kh.; Kokorin A. I. Rotational and Translational Mobility of Nitroxide Spin Probes in Ionic Liquids and Molecular Solvents. Appl. Magn. Reson. 2010, 39 (4), 409–421. 10.1007/s00723-010-0177-1.

[ref24] Kawai A.; Hidemori T.; Shibuya K. Polarity of Room-Temperature Ionic Liquid as Examined by EPR Spectroscopy. Chem. Lett. 2004, 33 (11), 1464–1465. 10.1246/cl.2004.1464.

[ref25] Evans R. G.; Wain A. J.; Hardacre C.; Compton R. G. An Electrochemical and ESR Spectroscopic Study on the Molecular Dynamics of TEMPO in Room Temperature Ionic Liquid Solvents. ChemPhysChem 2005, 6 (6), 1035–1039. 10.1002/cphc.200500157. 15937896

[ref26] Strehmel V.; Laschewsky A.; Stoesser R.; Zehl A.; Herrmann W. Mobility of Spin Probes in Ionic Liquids. J. Phys. Org. Chem. 2006, 19 (5), 318–325. 10.1002/poc.1072. 16596696

[ref27] Hwang J. S.; Mason R. P.; Hwang L. P.; Freed J. H. Electron Spin Resonance Studies of Anisotropic Rotational Reorientation and Slow Tumbling in Liquid and Frozen Media. III. Perdeuterated 2,2,6,6-Tetramethyl-4-Piperidone N-Oxide and an Analysis of Fluctuating Torques. J. Phys. Chem. 1975, 79 (5), 489–511. 10.1021/j100572a017.

[ref28] Freed J. H.; Fraenkel G. K. Semiclassical Theory of the Effects of Internal Motions on the Linewidths in Electron Spin Resonance Spectra. J. Chem. Phys. 1964, 41 (11), 3623–3638. 10.1063/1.1725777.

[ref29] Polimeno A.; Moro G. J.; Freed J. H. Rotational Dynamics of Axially Symmetric Solutes in Isotropic Liquids. I. A Collective Cage Description from Molecular Dynamics Simulations. J. Chem. Phys. 1995, 102 (20), 8094–8106. 10.1063/1.469220.

[ref30] Polimeno A.; Moro G. J.; Freed J. H. Rotational Dynamics of Axially Symmetric Solutes in Isotropic Solvents. II. The Stochastic Model. J. Chem. Phys. 1996, 104 (3), 1090–1104. 10.1063/1.470764.

[ref31] Pilar J.; Labský J.; Marek A.; Budil D. E.; Earle K. A.; Freed J. H. Segmental Rotational Diffusion of Spin-Labeled Polystyrene in Dilute Toluene Solution by 9 and 250 GHz ESR. Macromolecules 2000, 33 (12), 4438–4444. 10.1021/ma0002242.

[ref32] Stoesser R.; Herrmann W.; Zehl A.; Strehmel V.; Laschewsky A. ESR Spin Probes in Ionic Liquids. ChemPhysChem 2006, 7 (5), 1106–1111. 10.1002/cphc.200500651. 16596696

[ref33] Strehmel V.; Rexhausen H.; Strauch P. Influence of Imidazolium Bis(Trifluoromethylsulfonylimide)s on the Rotation of Spin Probes Comprising Ionic and Hydrogen Bonding Groups. Phys. Chem. Chem. Phys. 2010, 12 (8), 1933 10.1039/b920586a. 20145861

[ref34] Noel M. A. M.; Allendoerfer R. D.; Osteryoung R. A. Solvation in Ionic Liquids: An EPR Study. J. Phys. Chem. 1992, 96 (5), 2391–2394. 10.1021/j100184a070.

[ref35] Sengupta A.; Rm K. Electron Paramagnetic Resonance Spectroscopic Investigation of the Dynamics of Spin Probe in Room Temperature Ionic Liquid. Mod. Chem. Appl. 2016, 04 (04), 1000189 10.4172/2329-6798.1000189.

[ref36] Miyake Y.; Kawai A. Solvation and Rotational Diffusion of Solutes in Room Temperature Ionic Liquids as Studied by EPR Spectroscopy with Nitroxide Spin Probing Method. Appl. Magn. Reson. 2018, 49 (8), 825–835. 10.1007/s00723-018-1025-y.

[ref37] Miyake Y.; Akai N.; Kawai A.; Shibuya K. Hydrodynamic Interpretation on the Rotational Diffusion of Peroxylamine Disulfonate Solute Dissolved in Room Temperature Ionic Liquids As Studied by Electron Paramagnetic Resonance Spectroscopy. J. Phys. Chem. A 2011, 115 (24), 6347–6356. 10.1021/jp112151d. 21574586

[ref38] Hwang J.; Kivelson D.; Plachy W. ESR Linewidths in Solution. VI. Variation with Pressure and Study of Functional Dependence of Anisotropic Interaction Parameter, κ. J. Chem. Phys. 1973, 58 (4), 1753–1765. 10.1063/1.1679420.

[ref39] Davydov D. R.; Yang Z.; Davydova N.; Halpert J. R.; Hubbell W. L. Conformational Mobility in Cytochrome P450 3A4 Explored by Pressure-Perturbation EPR Spectroscopy. Biophys. J. 2016, 110 (7), 1485–1498. 10.1016/j.bpj.2016.02.026. 27074675 PMC4833771

[ref40] Lerch M. T.; López C. J.; Yang Z.; Kreitman M. J.; Horwitz J.; Hubbell W. L. Structure-Relaxation Mechanism for the Response of T4 Lysozyme Cavity Mutants to Hydrostatic Pressure. Proc. Natl. Acad. Sci. U. S. A. 2015, 112 (19), E2437–E2446. 10.1073/pnas.1506505112. 25918400 PMC4434698

[ref41] McCoy J.; Hubbell W. L. High-Pressure EPR Reveals Conformational Equilibria and Volumetric Properties of Spin-Labeled Proteins. Proc. Natl. Acad. Sci. U. S. A. 2011, 108 (4), 1331–1336. 10.1073/pnas.1017877108. 21205903 PMC3029758

[ref42] Gierer A.; Wirtz K. Molekulare Theorie Der Mikroreibung - Molecular Theory of Microfriction. Z. Für Naturforschung Sect. A 1953, 8, 532–538. 10.1515/zna-1953-0903.

[ref43] Mladenova B. Y.; Kattnig D. R.; Grampp G. Room-Temperature Ionic Liquids Discerned Via Nitroxyl Spin Probe Dynamics. J. Phys. Chem. B 2011, 115 (25), 8183–8198. 10.1021/jp201703c. 21634394

[ref44] Mladenova B. Y.; Chumakova N. A.; Pergushov V. I.; Kokorin A. I.; Grampp G.; Kattnig D. R. Rotational and Translational Diffusion of Spin Probes in Room-Temperature Ionic Liquids. J. Phys. Chem. B 2012, 116 (40), 12295–12305. 10.1021/jp306583g. 22928518

[ref45] Kundu K.; Kattnig D. R.; Mladenova B. Y.; Grampp G.; Das R. Electron Spin–Lattice Relaxation Mechanisms of Nitroxyl Radicals in Ionic Liquids and Conventional Organic Liquids: Temperature Dependence of a Thermally Activated Process. J. Phys. Chem. B 2015, 119 (12), 4501–4511. 10.1021/acs.jpcb.5b00431. 25775000

[ref46] Rasmussen K.; Hussain T.; Landgraf S.; Grampp G. High Pressure ESR Studies of Electron Self-Exchange Reactions of Organic Radicals in Solution. J. Phys. Chem. A 2012, 116 (1), 193–198. 10.1021/jp206464t. 22133086

[ref47] Stoll S.; Schweiger A. EasySpin, a Comprehensive Software Package for Spectral Simulation and Analysis in EPR. J. Magn. Reson. 2006, 178 (1), 42–55. 10.1016/j.jmr.2005.08.013. 16188474

[ref48] Lehner J.; Stoll S. Modeling of Motional EPR Spectra Using Hindered Brownian Rotational Diffusion and the Stochastic Liouville Equation. J. Chem. Phys. 2020, 152 (9), 094103 10.1063/1.5139935. 33480723 PMC7051866

[ref49] Ahosseini A.; Scurto A. M. Viscosity of Imidazolium-Based Ionic Liquids at Elevated Pressures: Cation and Anion Effects. Int. J. Thermophys. 2008, 29 (4), 1222–1243. 10.1007/s10765-008-0497-7.

[ref50] Carda-Broch S.; Berthod A.; Armstrong D. W. Solvent Properties of the 1-Butyl-3-Methylimidazolium Hexafluorophosphate Ionic Liquid. Anal. Bioanal. Chem. 2003, 375 (2), 191–199. 10.1007/s00216-002-1684-1. 12560962

[ref51] Huang J.-F.; Chen P.-Y.; Sun I.-W.; Wang S. P. NMR Evidence of Hydrogen Bonding in 1-Ethyl-3-Methylimidazolium-Tetrafluoroborate Room Temperature Ionic Liquid. Inorg. Chim. Acta 2001, 320 (1), 7–11. 10.1016/S0020-1693(01)00477-7.

[ref52] Huang J.-F.; Chen P.-Y.; Sun I.-W.; Wang S. P. NMR Evidence of Hydrogen Bond in 1-Ethyl-3-Methylimidazolium-Tetrafluoroborate Room Temperature Ionic Liquid. Spectrosc. Lett. 2001, 34 (5), 591–603. 10.1081/SL-100106873.

[ref53] Holbrey J. D.; Seddon K. R. The Phase Behaviour of 1-Alkyl-3-Methylimidazolium Tetrafluoroborates; Ionic Liquids and Ionic Liquid Crystals. J. Chem. Soc., Dalton Trans. 1999, (13), 2133–2140. 10.1039/a902818h.

[ref54] Miyake Y.; Hidemori T.; Akai N.; Kawai A.; Shibuya K.; Koguchi S.; Kitazume T. EPR Study of Rotational Diffusion in Viscous Ionic Liquids: Analysis by a Fractional Stokes–Einstein–Debye Law. Chem. Lett. 2009, 38 (2), 124–125. 10.1246/cl.2009.124.

[ref55] Köddermann T.; Ludwig R.; Paschek D. On the Validity of Stokes-Einstein and Stokes-Einstein–Debye Relations in Ionic Liquids and Ionic-Liquid Mixtures. ChemPhysChem 2008, 9 (13), 1851–1858. 10.1002/cphc.200800102. 18752221

[ref56] Kattnig B. Y. M.; Chumakova N. A.; Kattnig D. R.; Grigor’ev I. A.; Grampp G.; Kokorin A. I. Influence of the Electric Charge of Spin Probes on Their Diffusion in Room-Temperature Ionic Liquids. J. Phys. Chem. B 2021, 125 (32), 9235–9243. 10.1021/acs.jpcb.1c02493. 34378388

[ref57] Taylor A. W.; Licence P.; Abbott A. P. Non-Classical Diffusion in Ionic Liquids. Phys. Chem. Chem. Phys. 2011, 13 (21), 10147 10.1039/c1cp20373h. 21526251

[ref58] Vorotyntsev M. A.; Zinovyeva V. A.; Picquet M. Diffusional Transport in Ionic Liquids: Stokes–Einstein Relation or “Sliding Sphere” Model? Ferrocene (Fc) in Imidazolium Liquids. Electrochim. Acta 2010, 55 (18), 5063–5070. 10.1016/j.electacta.2010.03.070.

[ref59] Świergiel J.; Jadżyn J. Fractional Stokes–Einstein–Debye Relation and Orientational Entropy Effects in Strongly Hydrogen-Bonded Liquid Amides. Phys. Chem. Chem. Phys. 2011, 13 (9), 3911 10.1039/c0cp01900c. 21210040

[ref60] Einstein A. Zur Theorie der Brownschen Bewegung. Ann. Phys. 1906, 324 (2), 371–381. 10.1002/andp.19063240208.

